# Transcriptome Profiling, Physiological and Biochemical Analyses Reveal Comprehensive Insights in Cadmium Stress in *Brassica carinata* L.

**DOI:** 10.3390/ijms25021260

**Published:** 2024-01-19

**Authors:** Tinghai Yang, Biao Pang, Lizhou Zhou, Lei Gu, Hongcheng Wang, Xuye Du, Huinan Wang, Bin Zhu

**Affiliations:** School of Life Sciences, Guizhou Normal University, Guiyang 550025, China; 222100100455@gznu.edu.cn (T.Y.); 21010100406@gznu.edu.cn (B.P.); 21010100413@gznu.edu.cn (L.Z.); leigu1216@nwafu.edu.cn (L.G.); wanghc@gznu.edu.cn (H.W.); duxuye@gznu.edu.cn (X.D.)

**Keywords:** *Brassica carinata*, transcriptome, cadmium tolerance, physiological response

## Abstract

With the constant progress of urbanization and industrialization, cadmium (Cd) has emerged as one of the heavy metals that pollute soil and water. The presence of Cd has a substantial negative impact on the growth and development of both animals and plants. The allotetraploid *Brasscia. carinata*, an oil crop in the biofuel industry, is known to produce seeds with a high percentage of erucic acid; it is also known for its disease resistance and widespread adaptability. However, there is limited knowledge regarding the tolerance of *B. carinata* to Cd and its physiological responses and gene expressions under exposure to Cd. Here, we observed that the tested *B. carinata* exhibited a strong tolerance to Cd (1 mmol/L CdCl_2_ solution) and exhibited a significant ability to accumulate Cd, particularly in its roots, with concentrations reaching up to 3000 mg/kg. Additionally, we found that the total oil content of *B. carinata* seeds harvested from the Cd-contaminated soil did not show a significant change, but there were noticeable alterations in certain constituents. The activities of antioxidant enzymes, including catalase (CAT), superoxide dismutase (SOD), peroxidase (POD), and ascorbate peroxidase (APX), were observed to significantly increase after treatment with different concentrations of CdCl_2_ solutions (0.25 mmol/L, 0.5 mmol/L, and 1 mmol/L CdCl_2_). This suggests that these antioxidant enzymes work together to enhance Cd tolerance. Comparative transcriptome analysis was conducted to identify differentially expressed genes (DEGs) in the shoots and roots of *B. carinata* when exposed to a 0.25 mmol/L CdCl_2_ solution for 7 days. A total of 631 DEGs were found in the shoots, while 271 DEGs were found in the roots. It was observed that these selected DEGs, which responded to Cd stress, also showed differential expression after exposure to PbCl_2_. This suggests that *B. carinata* may employ a similar molecular mechanism when tolerating these heavy metals. The functional annotation of the DEGs showed enrichment in the categories of ‘inorganic ion transport and metabolism’ and ‘signal transduction mechanisms’. Additionally, the DEGs involved in ‘tryptophan metabolism’ and ‘zeatin biosynthesis’ pathways were found to be upregulated in both the shoots and roots of *B. carinata*, suggesting that the plant can enhance its tolerance to Cd by promoting the biosynthesis of plant hormones. These results highlight the strong Cd tolerance of *B. carinata* and its potential use as a Cd accumulator. Overall, our study provides valuable insights into the mechanisms underlying heavy metal tolerance in *B. carinata*.

## 1. Introduction

Due to ongoing urbanization and rapid industrialization, the presence of heavy metal pollutants, such as cadmium (Cd), copper (Cu), lead (Pb), and manganese (Mn), has emerged as a significant environmental concern [1]. Among these pollutants, Cd is particularly alarming as it is widely distributed in the environment in the form of divalent cations (Cd^2+^), posing urgent and serious threats [2]. Chemical speciation modelling of the rhizosphere solutions revealed that nearly all of the Cd was dissolved and distributed among the bioavailable Cd^2+^, Cl-complexed, and humates-complexed pools, with only small quantities of Cd adsorbed to K/Na-aluminosilicates. The slightly acidic pH range of 5.4–6.2 and the complexation with Cl^−^ and humates in the rhizosphere promoted the solubility of Cd and facilitated its transfer to plants [3]. Moreover, Cd has been shown to exhibit high mobility in soil–plant systems and accumulate in plants. The excessive uptake of Cd by plants can destroy the absorption and transport of essential elements, thus causing structural damage to chloroplasts [4]. Humans and other animals can be exposed to excessive Cd via contaminated food and water, which can lead to various diseases, including cancer, renal dysfunction, and lung dysfunction [5]. Consequently, understanding the tolerance mechanisms related to Cd stress in plants and finding effective methods to remove excessive Cd from contaminated soil and water are urgent issues at present [6].

The excessive accumulation of Cd can lead to the overaccumulation of reactive oxygen species (ROS), which can significantly reduce the photosynthetic capacity of plants. Consequently, this leads to increased lipid oxidation, leading to the inhibition of plant growth and development. The activities of antioxidant enzymes have been recognized as a reliable indicator of plant response to Cd stress. These enzymes, such as superoxide dismutase (SOD), glutathione reductase (GR), peroxidase (POD), and ascorbate peroxidase (APX), are capable of efficiently reducing the presence of reactive oxygen species (ROS) in plants [7]. In addition, proline (Pro) has been demonstrated to decrease lipid peroxidation and free radical levels, thereby preserving the integrity of the cell membrane in plants exposed to Cd stress [8]. The adsorption, translocation, and accumulation of heavy metals into plant cells generally depend on the following processes: binding and trapping to the root cell wall, transport into the cytoplasm, and sequestration into leaf vacuoles [2]. These processes are strictly regulated by a series of protein and transporter gene families [9]. Recently, several studies have demonstrated a positive association between some transporter genes from the *NRAMP* (genes of natural resistance-associated macrophage proteins) family and heavy metal uptake in rice, barley, and transgenic *Arabidopsis* [9,10]. Furthermore, it was observed that the accumulation of Cd in roots, branches, and seeds significantly declined after knocking out *NRAMP5* in rice. It is widely accepted that abiotic stresses, such as Cd stress, trigger the synthesis of many transcription factors, which regulate different signalling pathways and responses to these stresses in plants. WRKYs, a group of transcription factors in plants, have been shown to play a crucial role in regulating numerous biotic and abiotic stresses. Recent research conducted on various plants has demonstrated that the WRKYs can enhance Cd tolerance by controlling the expression of downstream target genes [11]. In *Arabidopsis*, *WRKY13* can activate the expression level of *PDR8* to positively regulate cadmium tolerance [12]. Another study by Jia et al. showed that *TaWRKY70* in wheat can positively regulate *TaCAT5* to strengthen Cd tolerance in transgenic *Arabidopsis* [13]. Moreover, research has indicated that heavy metal stress leads to an increase in the concentrations of abscisic acid (ABA) and ethylene (ETH), while the concentration of auxin (IAA) decreases. This suggests that genes associated with endogenous hormones respond to heavy metal stress [14].

Phytoremediation is widely recognized as an economical, efficient, and sustainable method for addressing heavy metal pollution in both soil and water, relying heavily on the ability of plants to absorb and retain these metals [15]. The Brassicaceae family, known as the mustard family, comprises 338 genera and more than 3700 species of flowering plants worldwide. This family not only contains many well-known commercial species, such as *Brassica* and *Raphanus* species but also hosts a multitude of heavy metal accumulators. Studies have revealed that approximately one-quarter of the hyperaccumulators identified so far belong to the Brassicaceae family, making it a suitable choice for studying the molecular mechanisms behind metal tolerance and hyperaccumulation [16].

Recent studies have shown the possibility of using some *Brassica. juncea* genotypes for potential phytoremediation and the mechanisms underlying the response of *Brassica. juncea* to Cd stress [17]. The allotetraploid *Brassica carinata* (BBCC 2n = 34), known as *Ethiopian rape*, was derived via natural interspecific hybridization between *B. nigra* (BB, 2n = 16) and *B. oleracea* (CC, 2n = 18). This species possesses numerous resistant traits to various biotic and abiotic stresses [18,19] due to its limited artificial domestication [20]. In addition, the seed oil of *B. carinata* is rich in erucic acid (40–45%), making it highly desirable as a biofuel and for industrial applications. This species can potentially be used as an ideal plant for the phytoremediation of Cd if it exhibits a strong ability in terms of Cd accumulation. However, the ability of *B. carinata* to accumulate Cd and its gene expression profile in responding to Cd have not been thoroughly investigated. This study will lay a theoretical foundation for improving its survival rate and yield in Cd-contaminated areas by exploring its tolerance to Cd at the seedling stage. To further understand the tolerance of *B. carinata* to Cd, the present study jointly employed phenotypic, physiological, and comparative transcriptomic approaches to detect its Cd accumulation capacity and gene expression patterns and to tap the genes associated with Cd stress to provide some molecular theoretical basis for understanding *B. carinata* in terms of Cd tolerance. The results showed that *B. carinata* had a strong tolerance to Cd and remarkable Cd accumulation ability, suggesting the possibility of using *B. carinata* as a Cd accumulator. Moreover, the total oil content of *B. carinata* seeds harvested from Cd-contaminated soil did not show a significant change. Physiological analysis demonstrated that some antioxidant enzymes responded quickly to Cd stress. The transcriptome results revealed a significant overrepresentation of transporter genes, particularly *WRKY* genes, and some endogenous hormone-related genes in the “Plant hormone signal transduction signalling” pathway, indicating their potential role in Cd tolerance. This study aims to analyze the cadmium tolerance of *B. carinata* through transcriptome and physiological and biochemical indicators. The findings will serve as a reference for further exploring the cadmium tolerance of Brassica plants.

## 2. Results

### 2.1. Cd Tolerance and Accumulation in B. carinata

**Figure 1:**
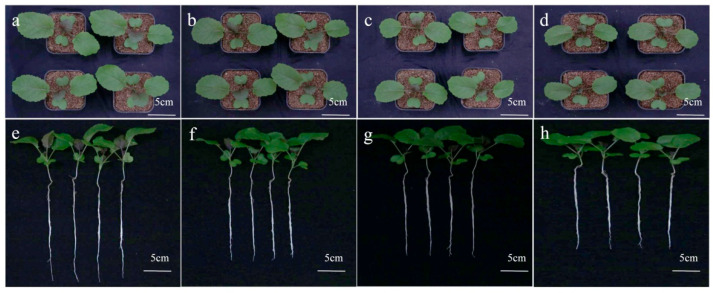
Seedlings performance of *B. carinata* exposed to different concentrations of CdCl_2_ solutions. (**a**–**d**) The performance of shoots of *B. carinata* plants treated with 0 mmol/L (CK), 0.25 mmol/L, 0.5 mmol/L, and 1 mmol/L CdCl_2_ solutions for 7 days. (**e**–**h**) The performance of young seedlings treated with 0 mmol/L (CK), 0.25 mmol/L, 0.5 mmol/L, and 1 mmol/L CdCl_2_ solutions for 7 days.

**Figure 2:**
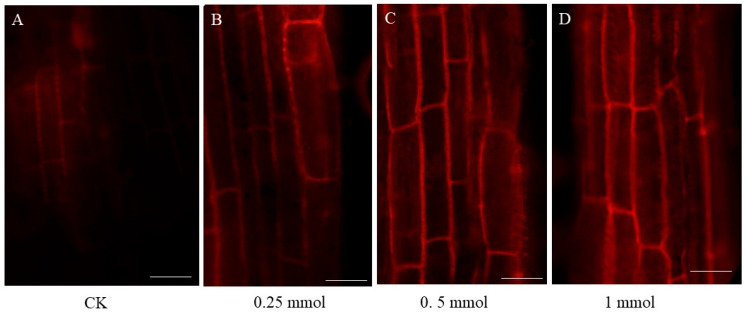
The changes in root microstructure after 7 days of different concentrations of Cd treatment. Bar = 50 μm. (**A**–**D**) The microstructure of *B. carinata* root tips in control group (**A**) and under different concentrations of Cd treatment (**B**–**D**) after PI staining.

*B. carinata* seedlings with three fully developed leaves were exposed to different concentrations of CdCl_2_ solutions (0.25, 0.5, and 1 mmol/L) for seven days. Compared to these controls, the treated seedlings exhibited noticeably delayed growth, with shorter roots and smaller leaves (Figure 1). In this study, we utilized the propidium iodide (PI) method to assess the integrity of *B. carinata* root tips following exposure to CdCl_2_ for seven days. The results showed that some PI staining solution entered the cytoplasm of root cells under the treatment of 1 mmol/L Cd; however, the cell membrane and cell wall remained largely undamaged at a concentration of 0.25 mmol/L and 0.5 mmol/L Cd, indicating the robust tolerance of *B. carinata* to low concentrations of cadmium (Figure 2). In particular, the biomass accumulation in the roots was seriously affected after treatment with 1 mmol/L CdCl_2_ solution; however, no obvious leaf defects were observed. Furthermore, there was no significant difference in the phenotypes of *B. carinata* seedlings after 7 days of treatment with Cd at concentrations of 0.25 mmol/L and 0.5 mmol/L, suggesting that *B. carinata* has a certain level of Cd tolerance.

**Figure 3:**
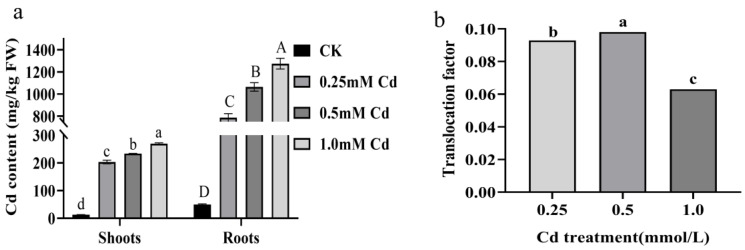
The changes in Cd content and translocation factor in shoots and roots after seven days of treatment with different concentrations of CdCl_2_ solution. (**a**): Cd content in shoots and roots. (**b**): translocation factor. Different letters represent the statistically different group, which is determined via multiple comparisons using Fisher’s least significant difference (LSD) method (*p* < 0.05). The error bar in the chart indicates the standard error (SE), and three replicates (*n* = 3) per sample are used to calculate the value of mean ± SE.

**Figure 4:**
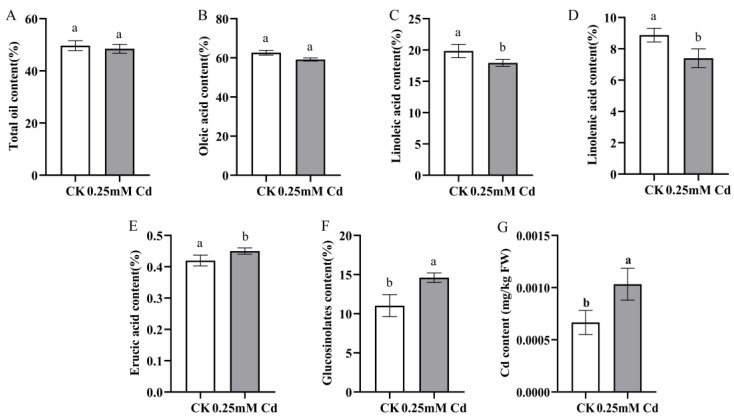
The oil content, constituents, and Cd content in seeds harvested from Cd-contaminated soil. (**A**): total oil content; (**B**): oleic acid content; (**C**): linoleic acid content; (**D**): linolenic acid content; (**E**): erucic acid content; (**F**): glucosinolates content; (**G**): Cd content. Different letters represent the statistically different group, which is determined via multiple comparisons using Fisher’s least significant difference (LSD) method (*p* < 0.05).

When exposed to a CdCl_2_ solution with a concentration of 0.25 mmol/L, the dehydrated roots and shoots of *B. carinata* contained 1805.72 ± 26.61 mg/kg and 168.32 ± 0.48 mg/kg of Cd, respectively. After exposure to CdCl_2_ solutions with concentrations of 0.5 mmol/L and 1 mmol/L, the dehydrated roots and shoots showed a significant increase in Cd content. The Cd content in the roots measured 2488.44 ± 95.10 mg/kg and 3022.38 ± 78.14 mg/kg, while the shoots had Cd content of 176.71 ± 3.65 mg/kg and 185.00 ± 0.89 mg/kg (Figure 3a; Table S1), respectively. Overall, *B. carinata* showed a strong capability in terms of Cd absorption but a relatively low delivery efficiency, with a Cd translocation factor (the ratio of shoot-to-root concentration) of only 0.093, 0.098, and 0.063 in the three treatments (Figure 3b), indicating the potential possibility of *B. carinata* developing high-Cd-absorption and accumulation crops. In addition, we measured the oil content and constituents in the *B. carinata* seeds harvested from harvested from Cd-contaminated soil (Cd content: 25 mg/kg). The result showed that, compared to the control group (CK), the total oil content did not exhibit a significant change (Figure 4A, Table S3), but there were noticeable alterations in certain constituents (Figure 4B–F), e.g., the levels of oleic acid decreased significantly, while erucic acid increased significantly. We also found that the Cd content in the seeds of the treatment group increased slightly compared with CK, but it was still lower than the national food standard (GB2762—2022) (0.5 mg/kg) (Figure 4G, Table S1) [21].

### 2.2. Physiological Responses of B. carinata to Cd Solutions of Varying Concentrations

**Figure 5:**
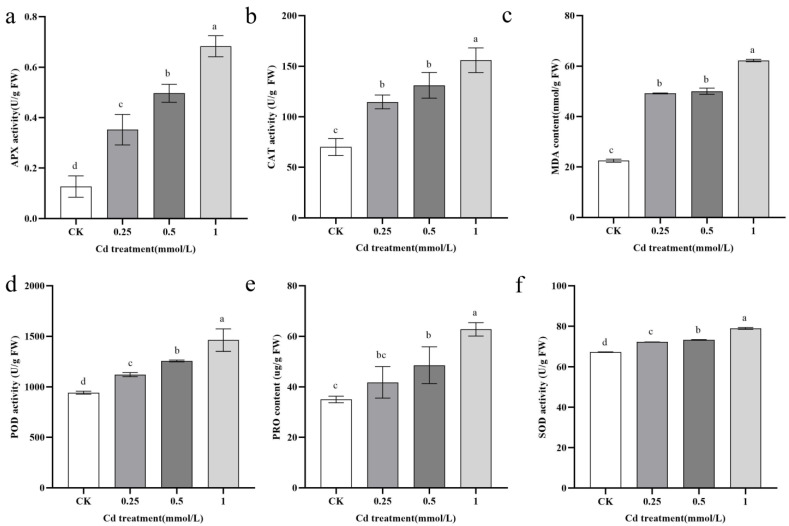
Physiological indicator changes in *B. carinata* seedlings under different concentrations of Cd treatment. (**a**): APX activity; (**b**): CAT activity; (**c**): MDA content; (**d**): POD activity; (**e**): PRO content; (**f**): SOD activity. Different letters represent the statistically different group determined via the LSD method (*p* < 0.05). The error bar in chart indicates SE, and three replicates (*n* = 3) per sample are prepared.

To ascertain the extent of physiological responses provoked by Cd stress in *B. carinata*, a range of antioxidant enzymes, such as SOD, POD, CAT, and APX, were evaluated alongside various physiological index content measurements, including Pro, MDA, and chlorophyll. These measurements were repeatedly conducted over a 7-day period under various Cd treatments. The findings indicate that exposure to various concentrations of CdCl_2_ had a significant impact on the levels of chlorophyll b (LSD test, *p* < 0.05). However, the 0.25 mmol/L CdCl_2_ treatment exhibited no significant difference compared to the control in terms of chlorophyll a and the total chlorophyll content (LSD test, *p* > 0.05). On the other hand, higher concentrations of CdCl_2_ resulted in a pronounced decrease in both chlorophyll a and total chlorophyll levels, with chlorophyll b demonstrating higher susceptibility to Cd stress (Figure S1). Other tested indicators showed a tendency of gradual decline with increased CdCl_2_ solution treatments (Figure 5). In brief, the MDA content significantly increased after treatment with CdCl_2_ solutions, especially with 1 mmol/L CdCl_2_ solution. The activities of APX, CAT, and POD were highly induced under Cd treatment (LSD test, *p* < 0.05). Specifically, treatment with 1.0 mmol/L CdCl_2_ resulted in a heightened APX activity of 0.683. This increase was also evident in Pro content, which was twice as much as that of the controls. Although SOD activity was enhanced after treatment with Cd, the increasing tendency was not obvious, similar to the abovementioned indicators.

### 2.3. Gene Expression Patterns in B. carinata in Response to Cd Stress

**Figure 6:**
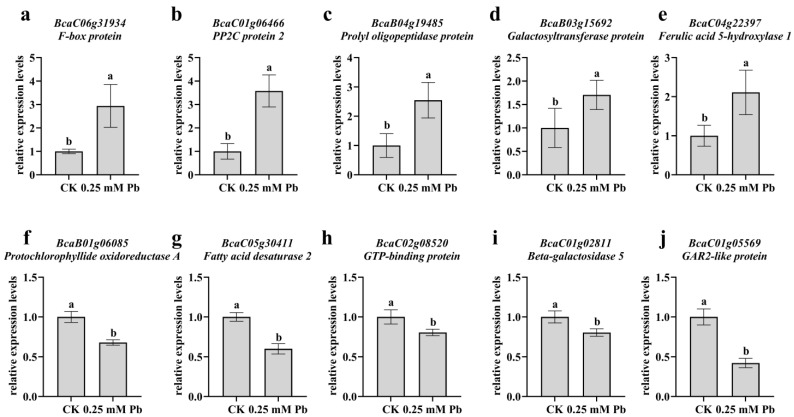
The expression patterns of differential genes in shoots after treatment with 0.25 mM PbCl_2_ solution for 7 days. (**a**–**e**): up-regulated DEGs. (**f**–**j**): down-regulated DEGs. The error bar in chart indicates SE, and three replicates (*n* = 3) per sample are prepared. Different letters represent the statistically different group determined via the LSD method (*p* < 0.05).

Transcriptome comparative analysis was conducted on seedlings treated with the control (CK) and 0.25 mmol/L cadmium chloride for 7 days to investigate the gene expression responses of the roots and shoots of *B. carinata* to Cd stress. After removing the adapter sequences and filtering out low-quality reads, a total of 79.16 Gb clean reads (ranging from 19,430,011 to 25,207,035 end-paired clean reads per sample) with a Q30 value of >91.05% were obtained from 12 c-DNA libraries. A total of 74.38–77.15% of these clean reads per sample were then mapped to *B. carinata*. A total of 2097 new transcripts were obtained after sequencing, of which 1460 new transcripts were functionally annotated using StringingTiev1.3.3 software [22]. The fragments per kilobase per million mapped fragments value (FPKM) of the gene was calculated to assess the gene expression level. In order to verify the precision of RNA-seq results, ten differentially expressed genes (DEGs) were selected randomly, comprising five upregulated and five downregulated DEGs. Their relative expression levels were assessed using qRT-PCR (Table S2). The results obtained from qRT-PCR were consistent with those from RNA-seq analysis (Figure S2), suggesting the reliability of the RNA-seq results. In addition, we treated the seedlings of *B. carinata* with 0.25 mmol/L PbCl_2_ solution for 7 days and measured these 10 DEGs relative expression patterns using qRT-PCR. It was observed that these selected DEGs, which responded to Cd stress, also showed differential expression after exposure to PbCl_2_ (Figure 6). This suggests that B. carinata may employ a similar molecular mechanism to tolerate these heavy metals. In comparison to CK, 631 DEGs were detected in the shoots (CKs vs. Cds) after exposure to Cd stress. Among these DEGs, 485 DEGs were upregulated (78.86% of the total difference), which was significantly higher than that of the downregulated DEGs (146 DEGs, 23.14%) (*χ*^2^-test, *p* < 0.01). In the comparison of CKr vs. Cdr, 271 DEGs (143 upregulated and 128 downregulated DEGs) were found, which was pronouncedly lower than that in CKs vs. Cds (*χ*^2^-test, *p* < 0.01). Based on the result of COG classification (Figure S3), the DEGs in CKs vs. Cds were mainly enriched in the following terms: “Signal transduction mechanisms”, “Secondary metabolites biosynthesis, transport and catabolism”, “Transcription”, “Carbohydrate transport and metabolism”, and “Defence mechanisms”. The DEGs in CKr vs. Cdr were primarily enriched in the following terms: “inorganic ion transport and metabolism”, “carbohydrate transport and metabolism”, “posttranslational modification, protein turnover, chaperones”, “general function prediction only”, and “secondary metabolite biosynthesis, transport and catabolism”. Moreover, only 22 DEGs were observed in both CKr vs. Cdr and CKs vs. Cds, suggesting that the gene expression response in the roots was quite different from that in the shoots under Cd stress.

### 2.4. GO Functions of the DEGs

**Figure 7:**
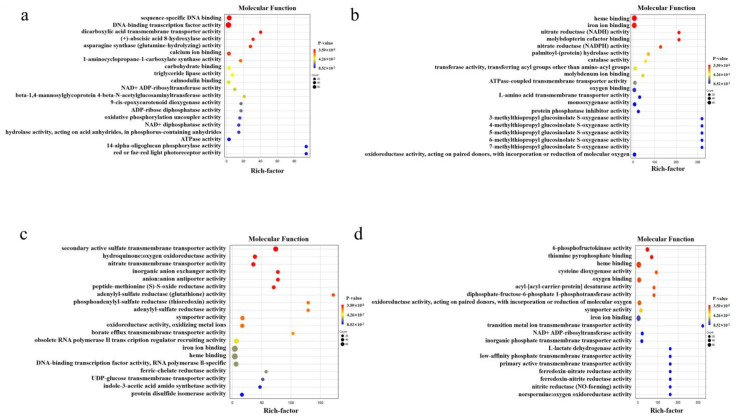
GO enrichment of DEGs in the comparisons of CKs vs. Cds and CKr vs. Cdr. (**a**,**b**): the upregulated and downregulated DEGs in CKs vs. Cds. (**c**,**d**): the upregulated and downregulated DEGs in CKr vs. Cdr.

To gain deeper insights into the roles of the DEGs induced by Cd stress, we conducted gene ontology (GO) analysis to explore their functional enrichment. The top 20 items enriched by these DEGs were selected and are listed in Figure 7. The results reveal that the upregulated DEGs in CKs vs. Cds were mainly enriched in “DNA-binding transcription factor activity”, “sequence-specific DNA binding”, “calcium ion binding”, “carbohydrate binding”, and “calmodulin binding” (Figure 7a), whereas the downregulated DEGs were significantly associated with functions like “heme binding”, “iron ion binding”, “oxygen binding”, “oxidoreductase activity, acting on paired donors, with incorporation or reduction of molecular oxygen”, and “monooxygenase activity” (Figure 7b). In the comparison of CKr vs. Cdr, the upregulated DEGs were mainly involved in “iron ion binding”, “heme binding”, “secondary active sulfate transmembrane transporter activity”, “obsolete RNA polymerase II transcription regulator recruiting activity”, and “DNA-binding transcription factor activity, RNA polymerase II-specific” (Figure 7c). On the other hand, the downregulated DEGs were abundant in “heme binding”, “iron ion binding”, “oxygen binding”, “oxidoreductase activity, acting on paired donors, with incorporation or reduction of molecular oxygen”, and “6-phosphofructokinase activity” (Figure 7d).

### 2.5. DEGs Enriched in KEGG Pathways

**Figure 8:**
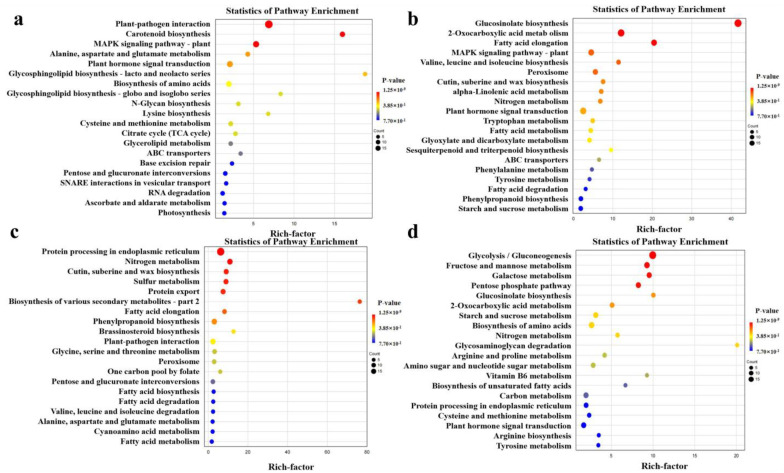
Top 20 KEGG enrichment pathways of DEGs in the comparisons of CKs vs. Cds and CKr vs. Cdr. (**a**,**b**): upregulated and downregulated DEGs in CKs vs. Cds. (**c**,**d**): upregulated and downregulated DEGs in CKr vs. Cdr. The *x*-axis represents the ratio of the number of enriched single genes (sample number) (based on the rich factor) to the number of annotated single genes (background number) in the path, and the *y*-axis represents the name of the pathway.

In addition, we utilized the Kyoto Encyclopedia of Genes and Genomes (KEGG) method to analyze the key genes responsible for Cd stress tolerance in *B. carinata*. The KEGG results (Figure 8) revealed that the upregulated DEGs in CKs vs. Cds were mainly enriched in pathways such as “Plant–pathogen interaction”, “MAPK signalling pathway-plant”, “Plant hormone signal transduction”, “Carotenoid biosynthesis”, and “Biosynthesis of amino acids” (Figure 8a), whereas the downregulated DEGs were remarkably involved in “Glucosinolate biosynthesis”, “2-Oxocarboxylic acid metabolism”, “Plant hormone signal transduction”, “Fatty acid elongation”, and “MAPK signalling pathway-plant” (Figure 8b). In the comparison of CKr vs. Cdr, the upregulated DEGs were primarily associated with processes such as “Protein processing in endoplasmic reticulum”, “Nitrogen metabolism”, “Phenylpropanoid biosynthesis”, “Cutin, suberine and wax biosynthesis”, and “Sulfur metabolism” (Figure 8c), but the regulated DEGs were abundant in “Glycolysis/Gluconeogenesis”, “Fructose and mannose metabolism”, “Galactose metabolism”, “Pentose phosphate pathway”, and “Biosynthesis of amino acids” (Figure 8d).

### 2.6. DEGs Involved in the Plant Hormone Signal Transduction Signalling Pathway

**Figure 9:**
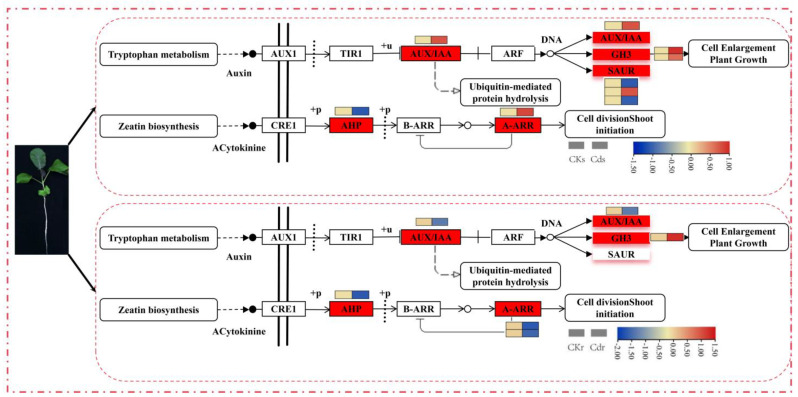
DEGs involving in the plant hormone signal transduction signalling pathway in both roots and shoots. The top-to-bottom heatmap representations correspond to the shoots and roots of *B. carinata*. The heat maps from left to right are CKs and Cds in shoots, and CKr and Cdr in roots.

We noticed that significant enrichments in terms of DEGs involved in the “Plant hormone signal transduction” pathway were detected in both comparisons of the CKs vs. Cds and CKr vs. Cdr. Therefore, we conducted a detailed analysis of these DEGs that were related to the “plant hormone signal transduction” pathway, with a particular focus on the genes associated with tryptophan metabolism and zeatin biosynthesis. As shown in Figure 9, nine DEGs involved in tryptophan metabolism and zeatin biosynthesis in the “plant hormone signal transduction signalling” pathway were observed in the shoots, of which six were upregulated genes, and three were downregulated genes. Among them, five upregulated genes (*BcaB07g028320*, *BcaB05g014490*, *BcaB05g052520*, *BcaB05g001550*, and *BcaB02g049160*) and two downregulated genes (*BcaB07g028900* and *BcaB02g089540*) were involved in tryptophan biosynthesis. Moreover, based on this pathway, these upregulated genes were mainly related to the expression of AUX/IAA and GH3 genes. In addition, two DEGs, including an upregulated (*BcaB02g036920*) and a downregulated (*BcaB05g000810*) gene involved in the biosynthesis of zeatin, were observed. In roots, three DEGs comprising an upregulated gene (*BcaB08g015910*) affect the synthesis of GH3, while the other two downregulated genes (*BcaB06g046620* and *BcaB04g019880*) relating to AUX/IAA conduction were found to be involved in tryptophan biosynthesis. In addition, three genes were involved in the biosynthesis of zeatin, including an upregulated gene (*BcaB07g028900*) involved in AHP transduction and two downregulated genes (*BcaB06g046620* and *BcaB06g000600*) affecting the expression of A-ARR.

### 2.7. Responses of Transcription Factors to Cd Stress in B. carinata

In this study, differentially expressed transcription factors altered by Cd stress were also detected in *B. carinata*. Intriguingly, the *WRKY* genes were likely susceptible to the effects of Cd stress, for which 26 and 6 *WRKY* DEGs were detected in the shoots and roots, respectively (Figure S4). Moreover, most of these WRKY DEGs (25 out of 26 in total) were upregulated in shoots under Cd stress, with only one downregulated gene. Similar results were observed in the roots, of which five genes were upregulated, and one gene was downregulated, indicating that these *WRKY* genes responded to Cd stress. In addition, three upregulated *WRKY* DEGs (*BcaB02g041040*, *BcaB05g041800*, and *BcaB08g042010*), orthologues of *Arabidopsis WRKY18*, *WRKY18*, and *WRKY40*, respectively, were detected in both roots and shoots. 

## 3. Discussion

To address the issue of Cd as a pollutant, it is crucial to identify plants that exhibit strong Cd tolerance and accumulation. Additionally, understanding the mechanisms behind the tolerance to Cd stress in these plants is essential [23]. Previous studies have shown that many species in Brassicaceae have outstanding Cd tolerance and accumulation abilities. As a *Brassica* species, allotetraploid *B. carinata* has not undergone artificial domestication. Thus, some desirable traits, particularly those resistant to various biotic and abiotic stresses, have been retained in *B. carinata* [24]. Here, we showed that *B. carinata* had a strong tolerance to Cd and remarkable Cd accumulation abilities. Although a small amount of Cd was detected in the control group (CK), this is likely due to the presence of trace amounts of Cd elements in the Hogland solution. The concentration of Cd in both the dehydrated roots and shoots exceeded the critical value for Cd hyperaccumulators [25] under different concentrations of Cd treatment. However, the Cd translocation factor (the ratio of shoot-to-root concentration) was found to be below 1.0 (the critical value for heavy metal hyperaccumulators) in *B. carinata*. This suggests that the excessive addition of Cd hindered the effective transport of Cd in a short period of time. A similar result was also observed in *Abelmoschus manihot* and *Erigeron canadensis* [8,22,26]. Although the results of this study were only based on hydroponic conditions, *B. carinata* showed a potentially strong ability in terms of Cd accumulation. Of course, further studies involving Cd-contaminated soil are necessary to confirm the phytoremediation ability of *B. carinata.*

In recent studies, it has been demonstrated that heavy metals can increase the toxicity of plants by leading to an overaccumulation of ROS, which in turn impairs the uptake and transportation of vital nutrients in plants [27]. When plants are exposed to abiotic stress, their ability to utilize light energy and assimilate carbon is hindered. Consequently, large quantities of ROS are produced in mitochondria, chloroplasts, and peroxisomes, which mainly contain superoxide anion (O_2−_), hydroxyl radical (-OH), and hydrogen peroxide (H_2_O_2_) [28]. Among them, excess O_2−_ causes lipid peroxidation and jeopardizes the cell membrane [29], and it will also produce more harmful ROS through the Mehler reaction; secondly, the large accumulation of H_2_O_2_ in plant cells will oxidize the thiol groups of some enzymes, which ultimately leads to the inactivation of some antioxidant enzymes involved in the plant body, and the hydroxyl radical (-OH) is the most active and harmful substance among ROS, as it significantly affects the affinity and oxidative reactivity of biomolecules at the site of generation [30,31].

Plants have developed a system of antioxidant enzymes, such as SOD, CAT, and APX, to combat the harmful effects of ROS [32]. SOD, being the most effective intracellular antioxidant, is found in various cell species susceptible to ROS. Its main role in plant resistance is to decrease the O_2_ content in the body, acting as the first line of defense against high levels of ROS toxicity [33]. CAT, on the other hand, converts H_2_O_2_ into H_2_O and O_2_, effectively reducing approximately 6 million H_2_O_2_ molecules per minute to water and O_2_. This enzyme plays a crucial role in mitigating the negative impacts of Cd stress and increases chlorophyll fluorescence parameters and photosynthetic pigment content [34]. APX is considered to be the most important enzyme in higher plants for scavenging reactive oxygen species to protect cells, and APX regulation under Cd stress is a postulated response. POD is a common oxidoreductase in plants, which can prevent the accumulation of hydroxyl groups in plants [32]. In addition, the increase in these antioxidant enzyme activities was also recognized as an important feature for plants as accumulators or superaccumulators [32]. In addition, Pro has been reported to scavenge excessive ROS caused by abiotic stress and is involved in the various metabolic activities of plants [35]. According to Sharma’s study, it was found that Pro has the ability to protect enzyme activity from heavy metals in vitro [36]. Specifically, Pro forms a non-toxic Cd-proline complex with Cd, effectively safeguarding Glucose-6-phosphate dehydrogenase. This enzyme plays a crucial role in the pentose phosphate pathway and is responsible for supplying plants with an ample amount of reduced glutathione (GSH). Additionally, it acts as a protective agent for sulfhydryl-containing proteins or enzymes, shielding them from oxidants [37]. In this study, an obviously increasing Pro content was observed under treatment with different Cd concentrations (Figure 5e) and similar results were observed for APX, CAT, and POD activities (Figure 5a,b,d). However, the SOD activity was not obviously increased under Cd stress, similar to the abovementioned indicators, indicating that *B. carinata* had certain detoxification to heavy metal cadmium stress.

In this study, we observed an overrepresentation of DEGs in the “tryptophan metabolism” and “zeatin biosynthesis” pathways, which are integral components of the “plant hormone signal transduction” pathway. This phenomenon was observed in both CKs vs. Cds and CKr vs. Cdr comparisons. Tryptophan plays a crucial role as a precursor for the synthesis of phyto-melatonin. Previous studies have reported that phyto-melatonin can enhance a plant’s ability to withstand adverse environmental conditions, thereby increasing its resistance to abiotic stress [38]. When plants are exposed to Cd stress, the genetic material DNA in their bodies is damaged to varying degrees. However, exogenous melatonin has been found to enhance stress tolerance by repairing DNA oxidative damage through various mechanisms, including RAPD polymorphism, DNA cross-link, 8-OH-dG level, the AP-site density, and an analysis of gene expression levels enriched in DNA damage repair pathways [33]. Studies have shown that melatonin can influence the gene expression of LOX, POX, and Asmap1 in the MAPK family of naked oat seedlings, which can be influenced by melatonin when exposed to Cd^2+^ stress. These studies have established the impact of melatonin on naked oat under Cd^2+^ stress conditions. Additionally, by enhancing the gene expression of *NACs* and *WRKYs* within the TFS family, melatonin stimulates the growth and development of naked oat seedlings, effectively intensifying their resistance to cadmium stress. In addition, the application of exogenous melatonin could significantly increase the contents of chlorophyll and proline in naked oat seedlings. Melatonin can also reduce the content of hydrogen peroxide, superoxide anion and malondialdehyde in naked oat seedling cells and increase the activity of SOD, POD and CAT [39,40,41]. Zeatin, a type of cytokinin, has been found to play a role in the response to Cd stress in *Arabidopsis thaliana*. It was observed that the endogenous CTK response gene CGA1 downstream signal transduction and nitrate-induced carbon metabolism genes GATA and CNC were stimulated and transcribed. CGA1 and GNC, as two major transcriptional regulators of chloroplast development, interact with each other to promote chloroplast division and growth [42,43,44]. Moreover, the activation of the cytokinin signal also contributes to the plant’s detoxification process. The plant itself releases nonprotein thiols such as glutathione, phytochelatins, and cysteine and complexes with heavy metals. The lower toxic complex is stored in the vacuole, reducing the migration of heavy metal ions and improving the tolerance of plants to cadmium [45]. 

WRKY TFs, also known as jack-of-all-trades, play a crucial role in regulating various developmental and physiological processes in plants. These processes include seed dormancy and germination, seed development, root formation, plant growth, senescence, trichome morphogenesis, and response to various biotic and abiotic stress factors [46]. Specifically, *WRKY18* and *WRKY60* interact with the W-box on the promoters of the genes such as *LCD*, *DCD1*, *DCD2*, *DES*, and *NFS2*, while *WRKY40* interacts with the W-box on the promoter of the *NFS1* gene [47,48,49]. The transcription factors *WRKY18*, *WRKY40* and *WRKY60* mainly act as inhibitors to regulate the transcription of genes that encode H_2_S synthase. H2S, in turn, maintains the redox balance by dynamically regulating NADPH oxidase and antioxidant enzyme systems, thereby preventing excessive apoptosis. The presence of H_2_S stimulates NADPH oxidase activity and enhances its ability to generate H_2_O_2_, both through the upregulation of transcription and enzyme activity. Similarly, H_2_S controls the levels of antioxidant enzymes, which also contributes to the reduction of reactive oxygen species contents. This improves the tolerance of plants to cadmium [50,51]. In this study, two *WRKY18* genes and one *WRKY40* gene were predicted, indicating that *WRKY18* and *WRKY40* genes may have a regulatory effect on *B. carinata* under Cd-induced stress. However, the specific mechanism of tolerance is still unclear and requires further investigation in future research.

## 4. Materials and Methods

### 4.1. Seedling Preparation and Experimental Conditions

A pure line of *B. carinata* with purple leaves, provided by Prof. Xianhong Ge from Huazhong Agriculture University, was used in this study. The seeds of this purple line were germinated in a sterile culture dish for sterile germination culture. Then, the plants were placed into black squares that contained vermiculite. The seedlings were raised in an incubator with the cultural parameters set as follows: a 16 h light cycle alternated with an 8 h dark cycle, a consistent temperature maintained at 22 °C, and a relative humidity level of 40%. A 1/2 Hoagland solution was applied to the seedlings every three days through spraying. After 10 days, 24 seedlings with similar growth were selected. Then, 500 mL of different concentrations of CdCl_2_ solution (0 mmol/L, 0.25 mmol/L, 0.5 mmol/L, and 1.0 mmol/L) prepared with 1/2 Hoagland solution was added to a 50 cm × 30 cm × 10 cm box and soaked for 7 days. In addition, the solution was changed every 2 days. To check the oil content and constituents affected by Cd, the seeds of *B. carinata* were placed in a Petri dish and grown in a 1/2 Hoagland nutrient solution for 14 days and then transplanted into a nutrient soil soaked containing 25 mg/kg of Cd to finish their entire life cycle. 

### 4.2. Propidium Iodide Staining

To check for the damage to root cells caused by Cd, the PI was dissolved and diluted with distilled water to a working solution (0.1 mg/mL). The samples were then placed on a slide, stained with PI working solution for 5 min and incubated in the dark for 20 min. Then, these tissues were rinsed using distilled water for 30 min. These tissues were finally observed using a fluorescence microscope (Nikon, N80i, Tokyo, Japan).

### 4.3. Determination of Cadmium Content in B. carinata

After being treated with CdCl_2_ solutions for seven days, a portion of both roots and shoots were frozen in liquid nitrogen and stored at −80 °C. These samples were selected for further physiological and transcriptome analyses. The rest of the seedlings were rinsed with tap water for 12 h, soaked in Na2-EDTA liquid (20 mmol/L) for 30 min, and then rinsed with sterile deionized water for half an hour. To determine the Cd content in the seedlings, a dehydration process was carried out in an oven set at 37 °C for three days to ensure the complete removal of moisture. 

The dehydrated sample was powdered and subjected to a series of treatments: first, it was first kept at 80 °C for 2 h in 70% nitric acid solution, then at 120 °C for 2 h, and finally at 160 °C for 4 h. After the nitrification treatment, it was cooled to room temperature. The content of heavy metal cadmium was determined using ICP-MS (Thermo X Series II, Nanjing, China).

### 4.4. Determination of Oil Content and Fatty Acid Content

The total oil content and the amount of oleic acid, linoleic acid, linolenic acid, erucic acid and glucosinolates in the seeds were determined through the use of near-infrared spectroscopy. The spectral acquisition conditions are as follows: a resolution of 8 cm^−1^, a scanning time of 64, and a spectral range of 12,000-4000 cm^−1^. The mathematical model between the predicted value and the standard chemical value was established through the use of the partial least squares method for optimization and verification.

### 4.5. Determination of Physiological and Biochemical Indices

Testing the activities of antioxidant enzymes in the examined *B. carinata* was conducted by using commercial kits specifically designed for physiological and biochemical indices (Beijing Soleibao Biotechnology Co., Ltd., Beijing, China), following the instructions provided. The MDA content was measured using a commercial kit according to the work of Hacer et al. [52]. The Pro content was processed with a commercially available testing kit following the guidelines of the manufacturer and assessed via a microplate reader at a UV wavelength of 520 nm. The method used to measure the chlorophyll content in the leaves was achieved through the extraction of acetone, as based on the work of Gan et al. [8]. The leaves were ground with liquid nitrogen (0.1 g) in the absence of light. Then, 1 mL of the chlorophyll extracting solution (acetone: anhydrous ethanol = 1:2) was added to the mixture. The mixture was thoroughly ground and placed in a dark environment to gently combine for 24 h so as to prevent any decomposition of chlorophyll. After centrifugation at 12,000× *g* for 10 min, the mixtures underwent chlorophyll level measurement using a microplate reader at UV levels of 663 nm and 645 nm.

### 4.6. Extracting RNA, Preparing cDNA Libraries, and Identifying Differentially Expressed Genes (DEGs)

The shoots and roots of the seedlings were collected and cryopreserved in liquid nitrogen after being exposed to a 0.25 mmol/L solution of CdCl_2_ for seven days. Three samples for the control (CK) and treated groups (Cd) were prepared. A commercial RNA extraction kit (EASY spin. Aidlad) was employed, following the instructions to extract total RNA from nearly 0.1 g of either the shoots or roots. To assess the quality of the extracted RNA, agarose electrophoresis was employed while the RNA integrity (RIN value) was measured using an Agilent 2100 instrument (Illumina, USA). To prepare the c-DNA library, we followed the TruSeq RNA Sample Prep v2 protocol (Illumina, USA), utilizing RNA RIN values of ≥8.0. A set of 12 c-DNA libraries was constructed and subjected to sequencing using the Illumina NovaSeq 6000 platform.

In this study, 150-bp paired-end reads were generated using the Illumina sequencing platform. To ensure the quality of the reads, Trimmomatic version 0.33 was utilized to eliminate reads containing adapters, poly-N, and low-quality bases. The high-quality reads, which were considered clean reads, were aligned to the newly published *B. carinata* reference genome [53] using Hisat2v2.0.5 with default parameters [54]. To ascertain the levels of gene expression, we utilized the FPKM values that were computed using RSEM’s default parameters. The differential expression genes between the untreated and treated *B. carinata* samples were identified using the R-project based on the Benjamini and Hochberg method (cut-off: *p* < 0.05 and fold changes >2). The raw sequence data can be accessed at NCBI-SRA (https://www.ncbi.nlm.nih.gov/sra, accessed on 24 May 2023) using the accession number PRJNA974582. 

### 4.7. Validation of the Sequencing Data by qRT–PCR

To ensure the accuracy of the RNA-seq sequencing data, we employed qRT-PCR to determine the relative expression level of genes in the aforementioned samples. A total of ten genes were randomly select for validation using qRT–PCR. The internal reference gene control for *B. carinata* adopted in this study was the actin gene. The SYBR-GREEN fluorescent reagent (TIANGEN Biotech, Beijing, China), comprising 10 μL 2 × SYBR ^®^ Premix Ex Taq II, 0.6 μL of forward primer, 0.6 μL of reverse primer, 2 μL of cDNA, and 6.4 μL of RNase-Free ddH_2_O, was used in this study. NCBI primer-blast technology was employed to design the primers for the chosen genes (Table S2).

### 4.8. Statistical Analysis

All of the experiments were performed using at least three technical replicates or three biological replicates. The data analyses were performed using IBM SPSS Statistics 26 and GraphPad Prism software (v: 9.0.0.121). The differences between various groups were analyzed using the LSD test.

## 5. Conclusions

After subjecting *B. carinata* to various concentrations of CdCl_2_ solution, it was observed that the plant exhibited a high level of tolerance toward cadmium and displayed significant accumulation of Cd. These findings indicate that *B. carinata* has the potential to be utilized as a Cd accumulator. Additionally, comparative transcriptome analysis showed that the plant hormone signalling pathway involved in tryptophan metabolism and zeatin synthesis, multiple antioxidant enzyme activities, and *WRKY* expression activity jointly responded to Cd stress in *B. carinata*. This study shows the possibility of using *B. carinata* for Cd phytoremediation and provides a reference for further exploring the cadmium tolerance of *Brassica species.*

## Data Availability

The raw sequence data can be accessed at NCBI-SRA using the accession number PRJNA974582.

## Supplementary Materials

The following supporting information can be downloaded at: https://www.mdpi.com/article/10.3390/ijms25021260/s1.
