# Correction: Yang et al. Transcriptome Profiling, Physiological and Biochemical Analyses Reveal Comprehensive Insights in Cadmium Stress in *Brassica carinata* L. *Int. J. Mol. Sci.* 2024, *25*, 1260

**DOI:** 10.3390/ijms26189091

**Published:** 2025-09-18

**Authors:** Tinghai Yang, Biao Pang, Lizhou Zhou, Lei Gu, Hongcheng Wang, Xuye Du, Huinan Wang, Bin Zhu

**Affiliations:** School of Life Sciences, Guizhou Normal University, Guiyang 550025, China; 222100100455@gznu.edu.cn (T.Y.); 21010100406@gznu.edu.cn (B.P.); 21010100413@gznu.edu.cn (L.Z.); leigu1216@nwafu.edu.cn (L.G.); wanghc@gznu.edu.cn (H.W.); duxuye@gznu.edu.cn (X.D.)

In the original publication [1], there were two mistakes in Figures 1 and 2, as well as one mistake in Figure S1. In Figure 1, we inadvertently duplicated the seedling performance of *Brassica carinata* exposed to 0.25 mmol/L CdCl_2_ and used it in place of the picture intended to represent 0.5 mmol/L CdCl_2_. As a result, both b and c in Figure 1 displayed the same phenotype. The authors also regret that in Figure 1, the image for 1h was mistakenly shown as a duplicate of 1g due to a figure assembly error. In Figure 2, the image for CK did not clearly show the root tip due to a figure select error, and we in-advertently duplicated the picture for 0.5 mmol/L CdCl_2_ and used it in place of the picture intended to represent 1 mmol/L CdCl_2_. As a result, both pictures displayed the same phenotype, and the actual picture for 1 mmol/L CdCl_2_ was omitted. In Figure S1, the graphs for chlorophyII-b content and total chlorophyII content were the same due to erroneously copying the images during figure assembly. As a result, both graphs in Figure S1 displayed the same chlorophyII-b content data, and the actual total chlorophyII content data was omitted. The corrected Figure 1, Figure 2 and Figure S1 and Legend are shown below. The authors state that the scientific conclusions are unaffected. This correction was approved by the Academic Editor. The original publication has also been updated.

## Figures and Tables

**Figure 1:**
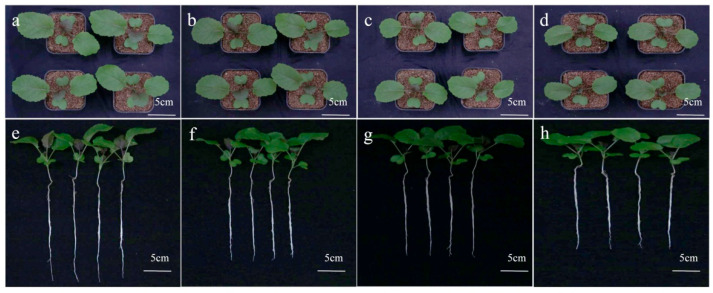
Seedlings performance of *B. carinata* exposed to different concentrations of CdCl_2_ solutions. (**a**–**d**) The performance of shoots of *B. carinata* plants treated with 0 mmol/L (CK), 0.25 mmol/L, 0.5 mmol/L, and 1 mmol/L CdCl_2_ solutions for 7 days. (**e**–**h**) The performance of young seedlings treated with 0 mmol/L (CK), 0.25 mmol/L, 0.5 mmol/L, and 1 mmol/L CdCl_2_ solutions for 7 days.

**Figure 2:**
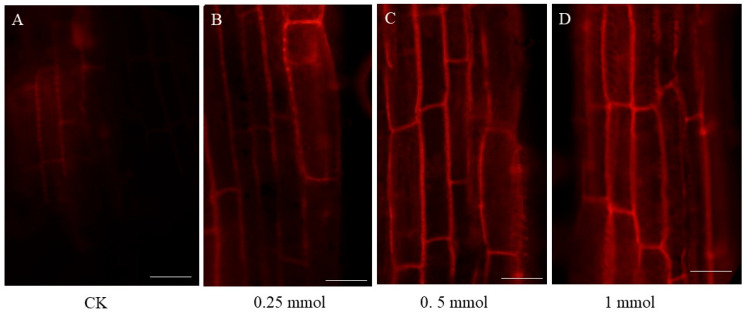
The changes in root microstructure after 7 days of different concentrations of Cd treatment. Bar = 50 μm. (**A**–**D**) The microstructure of *B. carinata* root tips in control group (**A**) and under different concentrations of Cd treatment (**B**–**D**) after PI staining.

**Figure S1:**
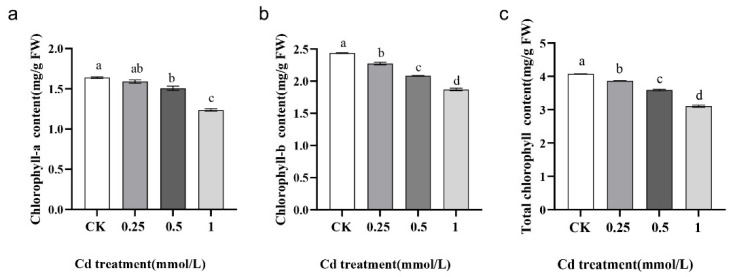
Chlorophyll content changes in *B. carinata* seedlings under different concentrations of Cd treatment. (**a**) Chlorophyll a content. (**b**) Chlorophyll b content. (**c**) Total chlorophyll content. Different letters represent statistically different groups determined by the LSD method (*p* < 0.05). The error bar in chart indicates the SE, and three replicates (n = 3) per sample were prepared.

## References

[1] Yang T., Pang B., Zhou L., Gu L., Wang H., Du X., Wang H., Zhu B. (2024). Transcriptome Profiling, Physiological and Biochemical Analyses Reveal Comprehensive Insights in Cadmium Stress in *Brassica carinata* L.. Int. J. Mol. Sci..

