# Supplementary material for: Transcriptome Profiling, Physiological and Biochemical Analyses Reveal Comprehensive Insights in Cadmium Stress in Brassica carinata L
Source: Int J Mol Sci. 2024 Jan 19;25(2):1260. doi: 10.3390/ijms25021260 (PMC10816673; doi:10.3390/ijms25021260)
Supplement: Supplementary file 1 [file ijms-25-01260-s001.zip › Supplementary Figures.pdf]

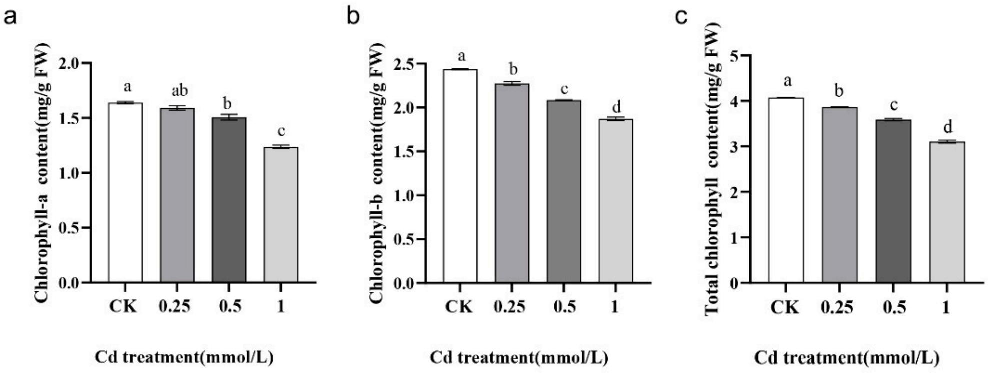

Figure S1. Chlorophyll content changes in *B. carinata* seedlings under different concentrations of Cd treatment. (**a**) Chlorophyll a content. (**b**) Chlorophyll b content. (**c**) Total chlorophyll content. Different letters represent statistically different groups determined by the LSD method ($p < 0.05$). The error bar in chart indicates the SE, and three replicates (n = 3) per sample were prepared.

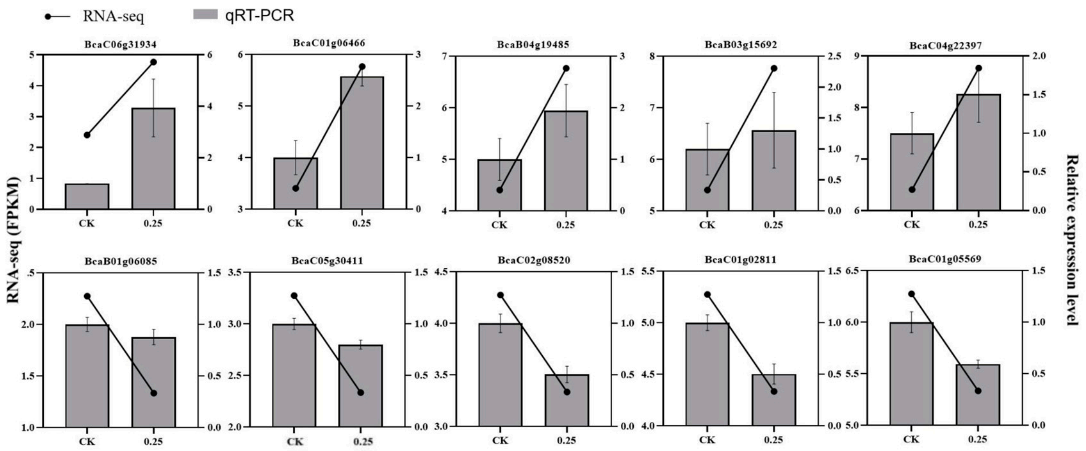

Figure S2. Validation of RNA-seq using qRT-PCR. The average FPKM value (black dot) and relative expression level (box) are shown in the figure. The error bar in chart indicates SE, and three replicates (n=3) per sample are prepared.

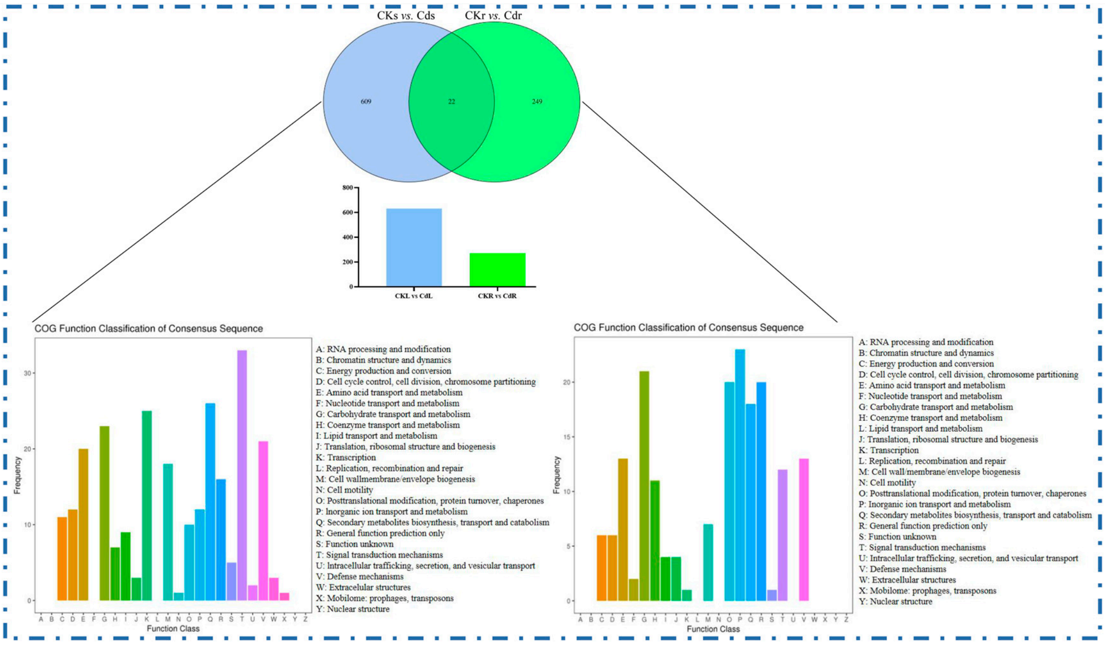

Figure S3. Venn diagrams and COG enrichment analysis of the DEGs in CKs *vs***.** Cds and CKr *vs*. Cdr.

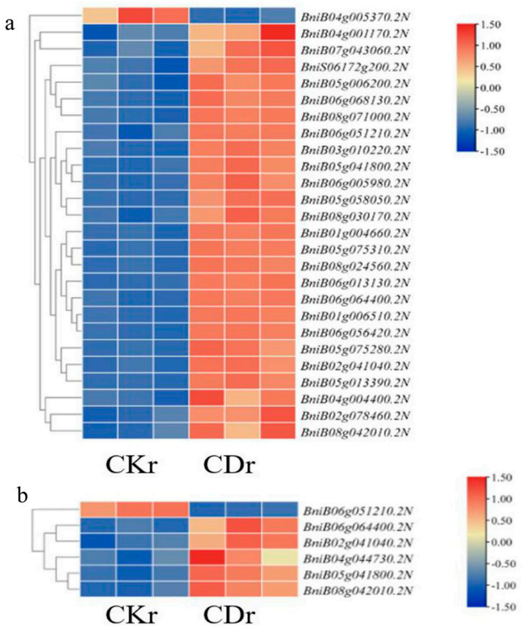

Figure S4. Clusters of differentially expressed *WRKY* genes in shoots and roots under Cd stress.
