# Supplementary material for: Transcriptome Profiling, Physiological and Biochemical Analyses Reveal Comprehensive Insights in Cadmium Stress in Brassica carinata L
Source: Int J Mol Sci. 2024 Jan 19;25(2):1260. doi: 10.3390/ijms25021260 (PMC10816673; doi:10.3390/ijms25021260)
Supplement: Supplementary file 1 [file ijms-25-01260-s001.zip › Supplementary Tables.pdf]

Table S1. Cadmium content of heavy metal after three days of treatment

Description of sample	Cadmium content (mg/kg)
0 mM Shoots	12.732
	12.414
	13.242
0.25 mM Shoots	196.685
	204.854
	209.233
0.5 mM Shoots	233.790
	233.708
	234.150
1 mM Shoots	272.160
	265.322
	270.273
0 mM Roots	50.618
	46.077
	51.047
0.25 mM Roots	766.880
	764.232
	829.865
0.5 mM Roots	1109.064
	1049.091
	1037.719
1 mM Roots	1318.424
	1222.559
	1285.277
0 mM Seeds	0.0008
	0.0006
	0.0006
0.25 mM Seeds	0.001
	0.0009
	0.0012

Table S2. Primers used in experiment of gene expression by qRT-PCR

Gene id	Forward primer	Reverse primer
actin	TTCAATGTCCCTGCCATGTA	GAGACGGAGGATAGCGTGAG
BcaC06g31934	CTCTCTCGTGTAAATTCCTTCG	CAGTGTTGCAAACCTTCCAA
BcaC01g06466	TGTATCAGTGGGAGTTGCGG	ATCAACGCTAATGCGGTCCA
BcaB04g19485	CGACCAAACAGGAGATGGGT	CCCATCCATCATCTCCCACG
BcaB03g15692	GCGTGGAGAAGGGTTGATGA	CCTCGTTAGCCACAAGCAGA
BcaC04g22397	TTCAGACGGGAGACTTTGGC	CGCGAGTGTTCTCCAGCTAT
BcaB01g06085	GCTTGGTGCATTGGTTCTGG	GCGGCAGCTTTTATCACGAC
BcaC05g30411	CTTGTTTTGGGAAGCCGTGG	ATCGGCATTGCGTTTGATCG
BcaC02g08520	CTACTCTGTTCGTGCAGCCA	TCTTGCACATGCTTCCACCA
BcaC01g02811	GACGGATAAAGGACGGCGAT	GGAAACGACGAAAGGCACAC
BcaC01g05569	ACTAGACTGGGGAGGCTACG	TCCGTGTGGTCACGAAGTTT

Table S3. Fatty acids and oil content

Description of sample	Oleic acid(%)	Linoleic acid(%)	linolenic acid(%)	Erucic acid(%)	Total oil content(%)	Glucosinolates(%)
CK1	63.74	18.91	8.49	0.38	50.05	10.02
CK2	61.32	21.00	9.35	0.36	51.36	12.62
CK3	62.78	19.61	8.79	0.36	47.64	10.44
0.25 mM Cd	58.35	17.53	8.09	0.39	50.36	15.26
0.25 mM Cd	59.93	18.57	7.09	0.40	48.01	14.46
0.25 mM Cd	59.18	17.76	7.01	0.40	47.00	14.08
